# Endocarditis caused by methicillin-susceptible *Staphylococcus aureus* with reduced susceptibility to vancomycin: a case report

**DOI:** 10.1186/1752-1947-5-292

**Published:** 2011-07-07

**Authors:** Beatriz Perazzi, Natalia Bello, Marta Mollerach, Carlos Vay, María Beatriz Lasala, Angela Famiglietti

**Affiliations:** 1 Clinical Bacteriology Laboratory. Department of Clinical Biochemistry. Hospital de Clinicas. Faculty of Pharmacy & Biochemistry. University of Buenos Aires. Córdoba 2351, Capital Federal. City of Buenos Aires. Argentina; 2 Division of Infectious Diseases. Hospital de Clinicas. Faculty of Medicine. University of Buenos Aires. Córdoba 2351, Capital Federal. City of Buenos Aires. Argentina; 3 Microbiological Laboratory. Department of Microbiology, Immunology and Biotechnology. Faculty of Pharmacy & Biochemistry. University of Buenos Aires. Junín 956, Capital Federal. City of Buenos Aires. Argentina

## Abstract

**Introduction:**

*Staphylococcus aureus *is the most common cause of acute infective endocarditis.

Recent reports have described heteroresistance to vancomycin associated with methicillin-resistant *Staphylococcus aureus*. We present the first case report in Argentina of the failure of treatment with vancomycin in endocarditis caused by methicillin-susceptible *Staphylococcus aureus *containing subpopulations with reduced susceptibility to vancomycin.

**Case presentation:**

We report the case of a 66-year-old Hispanic man with infective endocarditis complicated by septic emboli in the lumbosacral spine and the left iliopsoas muscle. This disease was caused by methicillin-susceptible *Staphylococcus aureus *containing subpopulations with reduced susceptibility to vancomycin. He was initially treated with cephalothin and gentamicin but developed a rash caused by beta-lactams and interstitial nephritis. For that reason, the treatment was subsequently switched to vancomycin but he failed to respond. The infection resolved after administration of vancomycin in combination with gentamicin and rifampin.

**Conclusion:**

Our case report provides important evidence for the existence of subpopulations of methicillin-susceptible *Staphylococcus aureus *that have reduced susceptibility to vancomycin which would account for treatment failure. Our case raises an alert about the existence of these strains and highlights the need to determine the vancomycin minimum inhibitory concentration of *Staphylococcus aureus *to screen for the presence of strains that have reduced vancomycin susceptibility at different infection sites.

## Introduction

*Staphylococcus aureus *is the most common cause of acute infective endocarditis (IE). *S. aureus *has developed resistance to every beta-lactam antibiotic that has been introduced into clinical medicine. Recent reports have described heteroresistance to vancomycin associated with methicillin-resistant *S. aureus *(MRSA) [1]. However, the scope and clinical significance of such isolates are yet to be completely defined.

We present the first case report in Argentina of the failure of vancomycin treatment for endocarditis caused by methicillin-susceptible *S. aureus *(MSSA) containing subpopulations with reduced susceptibility to vancomycin.

## Case presentation

We report the case of a 66-year-old Hispanic man with a history of diabetes, psoriasis, smoking, alcoholism, hospitalization in the previous year due to upper gastrointestinal bleeding (UGB), gastric ulcer and bacteremic lower limb cellulitis caused by MSSA, who received intravenous cephalothin for 14 days. The patient had not previously been exposed to glycopeptide antibiotics. At admission, he presented with a febrile syndrome and chills and complained of lumbar pain that had persisted for more than 20 days. On physical examination, the patient was mentally alert and had a blood pressure of 110/70 mmHg, a heart rate of 70 beats/minute and a respiration frequency of 18 beats/minute. A systolic murmur (grade 4/6) was detected in the aortic and mitral valves. He also had lower limb hypotrophia with no pain when flexing, extending or rotating the hip. A hematological study showed hematocrit levels of 28%, a hemoglobin concentration of 9.6 g/dL, a white blood cell count of 11,600/mm^3 ^with 85% polymorphonuclear leukocytes, a platelet count of 197,000/mm^3 ^and an erythrocyte sedimentation rate (ESR) of 130 mm in the first hour.

Blood cultures performed at admittance were positive for MSSA (SA1) in both samples taken after 18 hours. The SA1 strain was susceptible to the following non-beta lactam antibiotics: gentamicin, minocycline, tigecycline, rifampin, cotrimoxazol, vancomycin, teicoplanin, levofloxacin, ciprofloxacin and linezolid. The vancomycin MIC determined by the broth microdilution method was 1 μg/mL. Nuclear MRI of the spine showed spondylodiscitis at the L5-S1 level with left iliopsoas muscle involvement.

**Figure 1:**
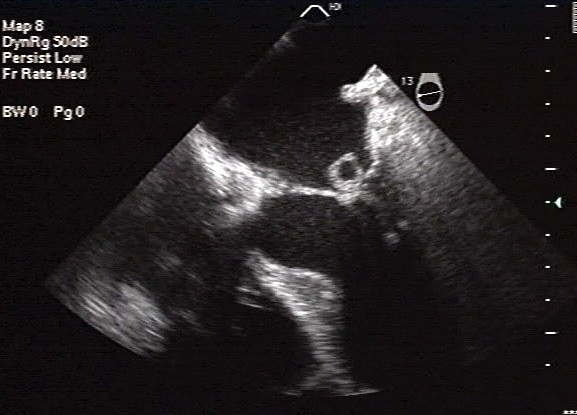
**Transesophageal echocardiogram**. A rounded mass, 1 cm in diameter, attached to the auricular side of the anterior mitral valve leaflet, compatible with vegetation and abscess, is observed. A smaller mass, 0.5 cm in diameter, is observed on top of the abovementioned mass, causing mild mitral failure.

The transesophageal echocardiogram (TEE) performed 48 hours after admission showed a mass compatible with vegetation and an anterior mitral valve leaflet abscess causing mild mitral failure (Figure 1): this was interpreted as IE with septic emboli involving the lumbosacral spine and the left iliopsoas muscle. Intravenous antibiotic treatment with cephalothin (2 g/6 hours) and gentamicin (240 mg/day) was started. Subsequent echocardiograms performed 15 days after the start of the treatment did not reveal any abscesses or changes in vegetation size. A blood culture performed as a control 10 days after the start of the treatment was negative.

On day 26 of cephalothin administration, our patient developed a rash caused by beta-lactams with eosinophilia and urinary sediment findings that were compatible with interstitial nephritis. For that reason, treatment was switched to intravenous vancomycin (1 g/12 hours) until day 42, when a new TEE was performed, which showed no vegetation. He was discharged due to improvement of his condition. However, a week later, a new blood culture was positive for MSSA (SA2) in both samples after 19.5 hours, showing the same antibiotic susceptibility and a vancomycin MIC of 1 μg/mL. He was readmitted 48 hours after the blood culture was performed. Immediate treatment was started with intravenous vancomycin (1 g/12 hours), gentamicin (240 mg/day) and rifampin (300 mg/6 hours).

A TEE performed 48 hours after hospitalization revealed a mass compatible with recently established aortic valve vegetation producing mild valvular failure. A mass attached to the anterior mitral valve leaflet was also observed, which suggested the presence of previously attached vegetation causing mild mitral failure. Our patient remained hemodynamically stable, afebrile and his physical examination was unremarkable. The treatment was monitored by a time-kill curve and by vancomycin dosage. The serum bactericidal rate showed bactericidal effects after 24 hours. The trough serum vancomycin concentration was 14.1 mg/L. Gentamicin was discontinued after 21 days due to renal failure. A TEE performed 20 days after hospitalization showed remission of the mass. A tomography-guided needle puncture of the lesion in the left iliopsoas muscle showed no microbiological growth.

Our patient completed intravenous treatment with vancomycin (42 days), gentamicin (21 days) and rifampin (36 days). Because the blood culture was negative, he was discharged.

Polymerase chain reaction detection of the *mecA *gene was negative in both isolates.

The vancomycin MIC for SA1 and SA2 with the standard inoculum (10^5^) was 0.5 and 1 μg/mL, respectively, and the minimum bactericidal concentration (MBC) was 0.5 and 128 μg/mL, respectively. A higher inoculum (10^7^) increased the MIC to 2 μg/mL and the MBC to 512 μg/mL in both isolates. After stimulation with increasing subinhibitory concentrations of vancomycin (SA3), the MIC and MBC with the standard inoculum were the same, whereas at a higher inoculum (10^7^), the values increased to 4 μg/mL and 512 μg/mL for the MIC and MBC, respectively.

**Figure 2:**
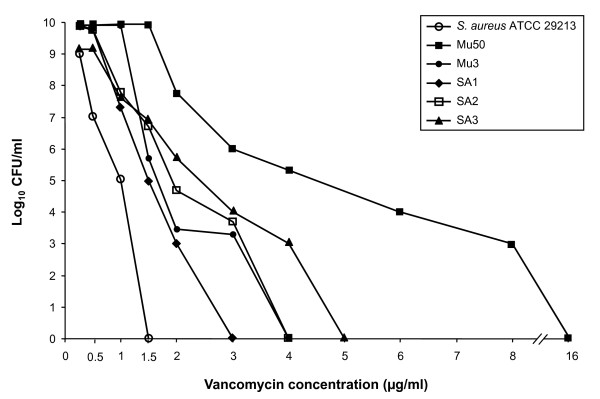
**Population analysis of isolates SA1, SA2, SA3, *S. aureus *ATCC 29213 (VSSA), *S. aureus *Mu3 (hVISA) and *S. aureus *Mu50 (VISA), assessed by susceptibility to vancomycin**.

In the population analysis of SA2 and SA3, a development of colonies up to 3 and 4 μg/mL, respectively, was observed, with growth between 1 and 4 μg/mL, which was 2 to 4 logs more than in SA1, which developed up to 2 μg/mL (Figure 2). SA2 and SA3 were identified as heterogeneous vancomycin-intermediate *S. aureus *(hVISA) on the basis of the population analysis profiling-area under the curve (PAP-AUC) ratios, showing PAP-AUC ratios of 1.06 and 1.26, respectively, compared to the AUC of the Mu3 strain, whereas SA1 was identified as vancomycin-susceptible, showing a PAP-AUC ratio of 0.83, compared to the AUC of the Mu3 strain [2].

**Figure 3:**
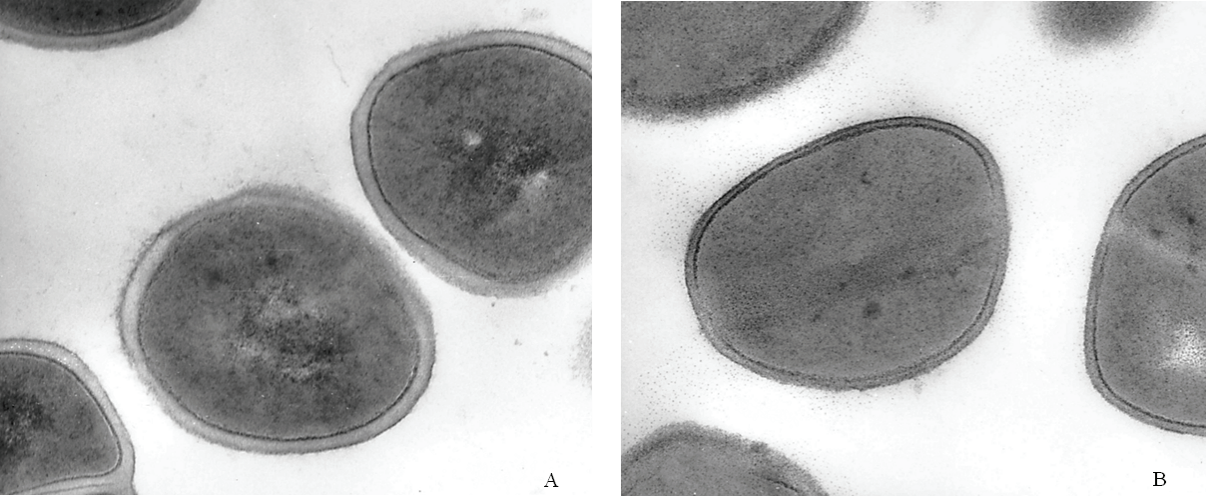
**Electron microscopy of the cell wall**. **A**. SA2, Cell wall thickness: 23.5 nanometers. **B**. Cell wall thickness of *S. aureus *ATCC 29213 strain: 15.6 nanometers.

Electron microscopy of SA2 showed a thickened cell wall (Figure 3).

The clonal relationship determined by pulsed-field gel electrophoresis showed that both *S. aureus *isolates displayed indistinguishable electrophoretic patterns.

## Discussion

IE is a disease in which the endocardial surface of the heart is invaded by infectious microorganisms. *S. aureus*, which is a common cause of acute IE, is difficult to treat and establishes an aggravated infection if the therapeutic options are limited because of adverse effects or reduced susceptibility to antibiotics.

IE due to *S. aureus *has a slow microbiological resolution when treated with vancomycin. This persistent bacteremia could be due to the presence of a metastatic infectious focus, such as that in the left iliopsoas muscle and the vertebrae. Because vancomycin has poor bone penetration, the initial monotherapy with this antibiotic combined with short-duration parenteral antimicrobial treatment may have failed to sterilize the bone. Negative cultures resulting from the aspiration biopsy of the muscle lesion cannot rule out the presence of a metastatic infectious focus, because this procedure has very low diagnostic sensitivity, especially in patients pre-treated with antibiotics. Another possible explanation of persistent bacteremia is that in this case, the isolate showed a vancomycin MIC ≥ 1 μg/mL, which could justify treatment failure and control of the infection with the combined treatment. In this respect, it is worth mentioning that treatment of MSSA bacteremia with vancomycin is not optimal, as has been clearly demonstrated in several studies. The slow bactericidal activity of this antibiotic is responsible for its high probability of therapeutic failure, which increases as the MIC increases, even within the susceptibility range [3]. There are several strategies to deal with this situation. The use of high doses of vancomycin (15-20 mg/kg/8 to 12 hours) in complicated infections to obtain trough serum concentrations of 15-20 mg/L and an AUC/MIC of > 400 has elicited a better therapeutic response in strains with MICs ≤ 1 μg/mL, despite higher rates of nephrotoxicity, which requires serum concentration monitoring of the drug [4]. The combination of vancomycin with other antibiotics, as in our case, is another possible strategy. Rifampin is a first-line anti-staphylococcal agent. However, some studies suggest that its combination with vancomycin may have antagonistic effects, although this was not the case with our patient [5]. Rifampin could have been effective here due to the patient's bone involvement in the spine. The combination of vancomycin with gentamicin was a previous recommendation of the Infectious Diseases Society of America and the American Heart Association to hasten clearance of blood cultures; this has recently been changed due to findings of enhanced nephrotoxicity with no real morbidity and/or mortality benefit [6].

An MIC of 1 μg/mL is not very frequent in *S. aureus *isolates. However, isolates with intermediate vancomycin susceptibility with MICs of 4-8 μg/mL (vancomycin-intermediate *S. aureus*: VISA, or glycopeptide-intermediate *S. aureus*: GISA) have been reported since 1997 [1,7-9]. Isolates which appear to be vancomycin-susceptible (MIC ≤ 2 μg/ml) but contain subpopulations expressing reduced susceptibility, known as heteroresistance (hVISA, or heterogeneous glycopeptide-intermediate *S. aureus*: hGISA), have been described [1,10-13]. These strains may exhibit vancomycin MICs of 1-2 μg/mL. Although the PAP-AUC method is considered the gold standard method for detection of hVISA strains, it is actually too time-consuming and labor-intensive for a clinical laboratory. Therefore, a new Etest hGISA/GISA resistance detection (GRD) strip (E-vancomycin/teicoplanin+supplement) recently validated in the US, has been described by Yusof et al. for detection of vancomycin heteroresistance [14]. The best performance for hGISA detection was found with the GRD strip on Mueller-Hinton blood with a sensitivity of 94% and a specificity of 95% at 48 hours, considering cutoff values of ≥ 8 for teicoplanin or vancomycin. The authors considered that the results for the GRD strip reading after 18 to 24 hours of incubation, if positive for hGISA/GISA, can be reported as such, although negative results should be confirmed after 48 hours of incubation because the sensitivity was highest at 48 hours [14]. This method has limited availability in Argentina.

The clinical impact of vancomycin treatment on these isolates is controversial. Musta et al. [15] compared the vancomycin MIC by Etest and the frequency of hVISA for all MRSA blood isolates and correlated the results with the clinical outcome, detecting hVISA in 30% and 80% of isolates with a vancomycin MIC of 2 and 3 μg/mL, respectively. An MIC of ≥ 2 g/ml was associated with a higher mortality rate. However, the vancomycin MIC and hVISA status did not affect mortality or persistent bacteremia. Bae et al. [16] characterized patients with IE using a multinational collection of isolates from MRSA with and without hVISA; they reported that patients with hVISA had a higher rate of persistent bacteremia and congestive heart failure but presented no differences in mortality from patients who were not infected with hVISA. hVISA isolates were genotypically similar to non-hVISA isolates. Maor et al. [17] compared patients who had hVISA bacteremia with those who had MRSA bacteremia. They reported that hVISA bacteremia was significantly associated with prolonged bacteremia duration, greater rates of complications such as endocarditis and osteomyelitis and emergence of rifampin resistance, compared with MRSA bacteremia. There was no significant difference in mortality between patients with hVISA bacteremia and those with MRSA bacteremia. Several authors have reported treatment failure with vancomycin in hVISA infections [9]. Moore et al. [18] described treatment failure with vancomycin in a patient with hVISA-associated endocarditis, as in our case. The isolate corresponded to a strain of MRSA, whereas in our case it was MSSA. In fact, unlike the organism observed in our case, most of the isolates described in the hVISA literature are MRSA. Nevertheless, Bobin-Dubreux et al. [19] in France reported a case of conjunctivitis due to MSSA in an hVISA isolate, and Fusco et al. [20] in the US also reported clinical failure of vancomycin in a dialysis patient with recurrent methicillin-susceptible vancomycin-heteroresistant *S. aureus *bacteremia. In addition, Pillai et al. [21] reported the development of reduced vancomycin susceptibility in a series of clinical methicillin-susceptible *S. aureus *isolates recovered from the blood and bone of a patient who experienced vancomycin therapy failure.

Different lines of evidence, such as population analysis and electron microscopy, suggest that vancomycin treatment failure of our endocarditis case could have occurred as a result of an hVISA infection. The fact that both *S. aureus *isolates had a clonal relationship suggests relapse and not reinfection. Considering that the vancomycin doses administered in our case did not reach the recommended trough serum levels of 15-20 mg/L (14.1 mg/L), it could be assumed that *S. aureus *subpopulations with reduced susceptibility to vancomycin might have arisen during therapy, thus contributing to treatment failure. The infection finally resolved after vancomycin treatment, likely because of its combination with gentamicin and rifampin.

Treatment failure of *S. aureus *endocarditis with other therapeutic alternatives, such as linezolid and daptomycin, have been reported [22,23]. Although clinical experience with daptomycin in *S. aureus *endocarditis is growing [24], the role of this antibiotic in the treatment of left-sided staphylococcal endocarditis is not clearly defined, and its availability in Argentina is limited.

## Conclusions

We describe the first case in Argentina of failure of vancomycin treatment in an acute infection caused by an hVISA methicillin-susceptible strain of *S. aureus*. Our report provides important evidence for the existence of subpopulations of *S. aureus *with reduced vancomycin susceptibility which would account for treatment failure in this case.

This case raises an alert about the existence of these strains, which despite showing vancomycin MIC values of ≤ 2 μg/mL, are considered susceptible by the Clinical and Laboratory Standards Institute (CLSI) [2]. These strains usually show vancomycin MIC values between 1 and 2 μg/mL which could account for treatment failure in severe infections if the trough serum concentrations of this antibiotic are lower than 20 μg/mL. Therefore, the correct management of severe *S. aureus *infections with vancomycin requires careful monitoring by determining the vancomycin MIC and its trough serum concentrations in order to adjust the treatment.

These findings raise awareness of the need to have an adequate screening method for the detection of vancomycin-heteroresistant strains that could be adapted to clinical laboratories in Argentina.

## Consent

Written informed consent was obtained from the patient for publication of this case report and accompanying images. A copy of the written consent is available for review by the Editor-in-Chief of this journal.

## Competing interests

The authors declare that they have no competing interests.

## Authors' contributions

NB and MBL performed clinical work, BP, MM, CV and AF carried out laboratory work, and all contributed to writing the article. All have read and approved the final manuscript.
