# Endocarditis caused by methicillin-susceptible *Staphylococcus aureus *with reduced susceptibility to vancomycin: a case report

**DOI:** 10.1186/1752-1947-5-555

**Published:** 2011-11-25

**Authors:** Beatriz Perazzi, Natalia Bello, Marta Mollerach, Carlos Vay, María Beatriz Lasala, Angela Famiglietti

**Affiliations:** 1 Clinical Bacteriology Laboratory. Department of Clinical Biochemistry. Hospital de Clinicas. Faculty of Pharmacy & Biochemistry. University of Buenos Aires. Córdoba 2351, Capital Federal. City of Buenos Aires. Argentina; 2 Division of Infectious Diseases. Hospital de Clinicas. Faculty of Medicine. University of Buenos Aires. Córdoba 2351, Capital Federal. City of Buenos Aires. Argentina; 3 Microbiological Laboratory. Department of Microbiology, Immunology and Biotechnology. Faculty of Pharmacy & Biochemistry. University of Buenos Aires. Junín 956, Capital Federal. City of Buenos Aires. Argentina

## Correction

Following the publication of our article [1] an error in the case presentation section was noted. In the description of the antibiotic susceptibility of SA1 strain, we incorrectly stated the value of vancomycin MIC for SA1.

The vancomycin MIC determined by the broth microdilution method was 1 μg/mL.

Should read:

The vancomycin MIC determined by the broth microdilution method was 0.5 μg/mL.
